# The advantage of DeepSeek in local deployment technology bridges the gap between research and practice

**DOI:** 10.1097/JS9.0000000000002784

**Published:** 2025-06-21

**Authors:** Chang JianBo, Xu HouShi, Wei JunJi

**Affiliations:** Department of Neurosurgery, Peking Union Medical College Hospital, Peking Union Medical College & Chinese Academy of Medical Sciences, Peking, PR China

## Abstract

**Figure:**
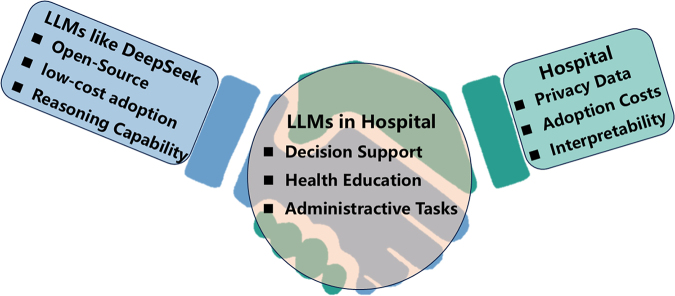

*Dear Editor,*

We read with great interest the studies by Sun *et al*^[1]^ on the potential applications of GPT in medicine. Although there have been many studies exploring the use of large language models (LLMs) to assist healthcare providers, and good results have been achieved in laboratory settings, there is a lack of experience in deploying these models in the real world. The main challenges between LLMs and clinical implementation include data privacy, adoption costs and interpretability.

DeepSeek^[2]^, as an advanced artificial intelligence technology, has demonstrated significant potential in transforming medical practices. This comment letter highlights the progress of LLMs like DeepSeek in tackling the technical pain points of clinical implementation, including open-source, low-cost adoption and reasoning capability. Understanding these technical features can help healthcare providers choose suitable LLMs that are easier to deploy in practice.

Firstly, data privacy and security are paramount in the medical field, and many hospitals perceive private, on-site deployment LLMs as a secure method for protecting patients’ privacy data. This requires LLMs to be open-sourced, allowing end users to fine-tune models to enable local training. From the perspective of open-source parameters, DeepSeek provides more permissions to modify the model. It offers access to its model parameters, weights, architecture, pre-trained model and partial source code. In comparison, ChatGPT and the Gemini series can only adjust API parameters, whereas LLaMA, Mistral, and Qwen have opened up their weights and partial code but have not made their pre-trained models and data publicly available. From the perspective of open-source licensing, DeepSeek adheres to the MIT Open Source License, which permits free commercial utilization and secondary development. ChatGPT and Gemini are primarily closed-source and require payment for use. Most versions of Mistral, Qwen, and LLaMA allow free commercial use, but large-scale enterprise use requires separate licensing. The first two follow the Apache 2.0 license, while the latter adheres to the Meta Community License. Currently, there is a trend toward more open-source LLMs. For example, Gemma, based on Gemini, provides model weights and follows the Apache 2.0 license. In summary, open-source LLMs that support more modifications can utilize privacy data, helping healthcare providers build derivative models more effectively on-site.

Secondly, the training and operational costs of LLMs are issues that hospitals need to take into consideration. Due to varying needs and private data, hospitals require low-cost local training. Compared to OpenAI’s ChatGPT, which relies on large-scale GPU clusters in the cloud, many lightweight versions of LLMs currently have lower hardware requirements. For instance, Mistral Small 3.1-24B and LLaMA-13B can be deployed on a single RTX 4090 (24GB). Meanwhile, DeepSeek achieved notable reductions in computational demand, its full version (DeepSeek R1-671B) just required eight A100 GPUs. In addition to training costs, the real-time response capability of LLMs is also a key factor for the deployment in real-world. The delays can have serious consequences in clinical scenarios. Unlike cloud-based services that may experience latency issues, LLMs’ local deployment ensures that response speed is unaffected by network conditions. The full version of DeepSeek (R1-671B) achieves 28 tokens/s with minimal computational resources (8 A100 GPUs). The lightweight versions of Mistral (Small 3.1-24B) achieves 150 tokens/s with 4 H100 GPUs. Above all, for LLMs, high performance with low computational power requirements is important in practice. The “low-cost” adoption makes it accessible to a broader range of medical facilities, thereby facilitating the widespread adoption of AI-driven solutions in healthcare and promoting the digital transformation of the medical industry.

Lastly, clinical decision-making requires integrating the best evidence, clinical expertise and patient values. Logical reasoning forms the foundation of decision-making. The interpretability of LLMs is key to helping physicians assess whether decisions are reasonable. DeepSeek enhances its reasoning ability through reinforcement learning. Before generating the final answer, DeepSeek produces a reasoning process for the question. And the reasoning steps provided by DeepSeek were deemed more accurate than those provided by ChatGPT and Llama 3.1-405B^[3]^.

The primary challenge faced by DeepSeek is model hallucinations^[4]^. “hallucinations” are often defined as LLMs generating that appear logically coherent yet are factually incorrect. This phenomenon is particularly prevalent in and arises due to limitations in training data or the model’s incomplete comprehension of the given context. Therefore, local training datasets based on clinical cases and guidelines can help reduce hallucinations in LLMs. Meanwhile, the RAG (Retrieval-Augmented Generation) technology of LLMs is also crucial, as it helps doctors trace the source of the answer and evaluate its reliability to avoid catastrophic mistakes.

In conclusion, DeepSeek has played a “catfish effect” role in bridging the gap between real world and LLMs. An increasing number of LLMs are becoming more open-source, focusing on adoption costs and reasoning capability. In China, as of 9 March 2025, more than 300 hospitals have adopted private, local, on-site deployments of DeepSeek^[5]^. DeepSeek is integrated into hospital information system (HIS) to assist doctors in discharge summaries, decision support, and health education. This comment letter might help healthcare providers learn how to choose a suitable LLM in practice in the future.

The letter is compliant with the TITAN Guidelines 2025^[6]^. We used generative AI to polish the draft, which does not involve factual content and has no conflicts of interest.

## Data Availability

This is a comment on a published paper; no data was presented in our comment, so the data statement is not applicable.
